# Comment on Pashchanka, M. Conceptual Progress for Explaining and Predicting Self-Organization on Anodized Aluminum Surfaces. *Nanomaterials* 2021, *11*, 2271

**DOI:** 10.3390/nano13212876

**Published:** 2023-10-30

**Authors:** Silvio Heinschke, Jörg J. Schneider

**Affiliations:** Eduard-Zintl-Institut für Anorganische und Physikalische Chemie, Technische Universität Darmstadt, Peter-Grünberg-Str. 12, 64287 Darmstadt, Germany

## 1. Introduction

In the review article “Conceptual Progress for Explaining and Predicting Self-Organization on Anodized Aluminum Surfaces” [1], the author Mikhail Pashchanka (MP) aims to give a thematic overview in the field of aluminum-metal anodization. Therein, a special focus is on theoretical concepts including electroconvection.

In the review, our paper “On the Prediction of Well Ordered Porous Anodic Alumina Films” [2] is discussed at length and severe criticism is raised towards its scientific content. However, a critical analysis of the review reveals shortcomings in the scientific reasoning against our theoretical approach. Therefore, herein, we comment on the relevant points to foster a scientific discussion.

## 2. Comment

The overall aim of our 2020 paper ([2], hereafter referred to as “our paper”) was to show that the porosity number p or P (lowercase letter in our paper and in [3]; uppercase letter in MP’s review paper [1]) can be derived from a model system based on irreversible thermodynamics and is, therefore, connected to the entropy production of the studied electrochemical system, as explained in our paper. For reasons of comprehensibility, p is given by

(1) $$p=\frac{q_{av}\Delta U}{\eta \sigma }$$

with $q_{av}=\frac{10^{-pH}}{C}$; where *C* is the electrolyte concentration, ΔU is the applied voltage, η is the dynamic viscosity and σ is the specific electric conductivity.

This aim of our paper was seemingly not part of MP’s reasoning, because he wrote that we “*claim the universality and general validity*” and assert that our theory “*provides an accurate description of the experimental reality*” (see [1] p. 49). In fact, we never claimed this in our paper. Instead and in contrast to the statement by MP, it was originally mentioned by us in our paper that as the porosity number p is a result of our derivation, the latter could reflect experimental reality since the validity of p was experimentally proven by MP himself in a joint paper together with one of current authors Jörg J. Schneider [3]. Hence, the result of our theory is, therefore, an experimentally proven parameter, which confirms our arguments and ensures comparability to the experiment. Therefore, there is no unjustified claim of universality or generality by us.

In this context, MP states that the criterion

(2) $$P_{eq,+}=2\frac{\Delta U}{\eta \sigma }\frac{I}{C}$$

where *I* is the ionic strength, which was derived by us (MP uses the definition *P* for $P_{eq,+}$), “*should better reflect its non-constant character*” (see [1] p. 49 Equation (6)). In fact, we never wrote this about $P_{eq,+}$ but instead stated that “*$P_{eq,+}$ is a more appropriate parameter for describing PAOX growth than p, because it is related to ionic strength*” (see our paper p. 11920 resp. p. 11919 Equation (54)). In contrast to MP’s statement, the non-constant character of p is a direct consequence of its relation to entropy production in our theory (see our paper p. 11919 conclusions).

Following his text further on the same page, MP identifies “*an apparent confusion between isothermal and adiabatic conditions*” in our theoretical approach. He draws his conclusion from our argument “*…with a defined constant temperature T. Thus, the system is isolated against heat exchange with the environment*” (see our paper p. 11914). Here, MP’s conclusion is based only on a part of the definitions made by us. Due to our theory, the isolation of the system against heat exchange goes along with the assumption that the entropy gain of the system by heat exchange is 0 ($\frac{d_{en}S}{dt}=0$). The entropy gain by heat exchange is given by $\frac{d_{en}S}{dt}=\frac{1}{T}\frac{dQ}{dt}$ [4]. The experimental setup consists of a cryostat, which ensures a constant temperature. To do so, the cryostat compensates the heat flow between the bath and its environment (hence, the heat flow between the bath and the atmosphere does not contribute to the formation of the anodic alumina film). The studied system (which possesses the intrinsic entropy production $\frac{d_{in}S}{dt}$) consists of the volume between the electrodes ($T_{system}$,$Q_{system}$), which is surrounded by the electrolyte bath ($T_{bath}$, $Q_{bath}$). As the system is assumed to have a constant temperature (as well as the surrounding bath), it follows that $0=\Delta T=T_{system}-T_{bath}\propto \Delta Q=\Delta Q_{system}-\Delta Q_{bath}=0$ because bath and system possess the same entropy ${}_{en}S$. As the temperature gradient between system and bath is 0, the heat exchange between bath and system is 0 (see [1] p. 7) and the system is isolated against a heat exchange with the environment, which is the atmosphere around the bath (see our paper pp. 11914 and 11915). Additionally, the term adiabatic used by MP does not apply to the experimental situation defined in our paper, because even in the case of the total isolation of the system against heat exchange, the definition “is held at constant temperature” (see our paper p. 11915) refers to the point that the bath has to be kept at the considered temperature. In that way, the generated heat is compensated. In reality, this is normally done by a cryostat, which exchanges the heat from the solution, which is created during the process, with the environment. In that way, the cryostat increases the entropy of the environment. In addition, the system where the irreversible process occurs experiences an entropy loss due to the evolution of an organized pattern (see [5] p. 136). However, this issue was not discussed in our paper because the focus was on the description of $\frac{d_{in}S}{dt}$.

Along with this, MP states that setting the condition that the bath is held at a constant temperature along with $\frac{d_{en}S}{dt}=0$ eliminates “*the critical factor that enables the formation of dissipative structures (i.e., self organization) as such*” (see [1] p. 49). This argument is in accordance with a statement by MP that he gives on p. 6 of his review: “*However, we should remember that self-organization always occurs in open systems with heat elimination*”. Here, it must be mentioned that the latter argument of MP was part of a text passage in which he elaborates on the thermodynamic factors that have to be taken into account to construct a cell for PAA synthesis. However, from the experimental point of view, these statements are difficult to substantiate scientifically, as indicated by well known self-organized structures and systems like in the Belousov–Zhabotinsky reaction, in the Turing pattern [5] or by the existence of the Marangoni Effect [6], which either depend on the non-linear response of a system to concentration differences or are induced by surface tension gradients.

From the theoretical point of view, MP’s statements about heat elimination given above are also not generally applicable, as can be seen, e.g., by arguments of Prigogine (see therefore [7] p. 66) or in the general textbook of Prigogine and Kondepudi, which address the theory of dissipative structures in a mathematical way [5]. Therein, it is shown that it is rather the production of entropy by an irreversible process induced by a specific driving force (i.e., a gradient), which is an inevitable condition for self-organization [5].

In the theory delineated in our paper, this driving force is represented by the electrochemical potential gradient, which transforms into an electrical potential gradient after ruling out other forces by mathematical means and suitable assumptions (see our paper p. 11917). Here, it is worth mentioning that the importance of the potential gradient has been already adressed in the original paper by MP and Jörg J. Schneider [3]. They wrote in their conclusions in [3]: “*While the temperature gradient is the driving force for conventional Rayleigh–Bénard cells’ spatial distribution, the applied voltage acts as a driving force for PAOX pattern formation*”. From this point of view, this shows that the mathematical definition of p and the associated chemical model do not necessarily correlate with each other. These facts where not discussed by MP.

In the light of these arguments, it is worth mentioning that MP states that “*Pashchanka theory served as a starting point for the authors’ theorizing*” (see [1] p. 50), which is to be doubted since “*Pashchanka theory*” (in form of the factor p) is the conclusion drawn from our theory and his assumptions (see our paper p. 11915 point (i)–(viii)). In advance, parts of MP’s original suggestions about the chemical mechanism of PAOX formation [1,3] is mathematically directly disproved by our paper, as it was shown that the diffusion terms of the Nernst–Planck equation are negligible.

In this context, MP presents a text passage by Prigogine (see below) with which he tries to underpin his statement and he seemingly interprets it as one of general validity:

“*The entropy produced by the irreversible heat flow leads to an ordering process, which would be impossible if taken independently from the heat flow*” ([7] p. 26).

However, in the original context, this is not the obvious intention of the author Prigogine, but rather the citation is taken out of its broader context in which Prigogine made his statement. This becomes fully evident if one studies Prigogine’s original text on p. 26 in [7] in its full meaning. Here, Prigogine elaborates on the possibility of the occurrence of order and disorder evolution in systems with thermal diffusion as an example, not in the general case of dissipative structure formation. However, there is no such statement by Prigogine in the context of this example that heat exchange is a “*…critical factor that enables the formation of dissipative structures (i.e., self organization) as such*”, which is what MP wrote in his review.

In sharp contrast to the presentation of Prigogine’s scientific reasoning by MP, Prigogine clearly depicts his ideas about the condition for the occurrence of dissipative structures in the same book (see [7] p. 66):

“*Thermodynamics leads us to the formulation of two conditions for the occurrence of dissipative structures in chemistry: (1) far-from equilibrium situations defined by a critical distance; and (2) catalytic steps, such as the production of the intermediate compound Y from compound X together with the production of X from Y*”.

In addition, the mentioned quote from Prigogine is not correctly cited by MP (cited page exceeds the number of pages in the book by Prigogine).

Furthermore, it was shown in our paper that the derivation of the parameter p necessarily requires that a stationary constant current after a specific time point $t_{c}$ evolves. Due to this, MP argues that the “Heinschke–Schneider-model” (a term created by MP) seemingly contains a fundamental flaw “*because it relies on the very specific parameter $t_{c}$, which in fact does not exist in a generic experimental situation*” (see [1] p. 50). It is a fact that the existence of a shift from non-stationary to stationary or steady state during an anodic alumina reaction is usually assumed or defined in the literature to be represented by the simple growth of the anodic film [8]. This can be found even in the papers that showed very different kinds of i–t curves, some of which MP cited (see [3,9,10,11,12]). From the theoretical point of view, $t_{c}$ does not necessarily have to be identifiable via the i–t curve as the assumption of the existence of $t_{c}$ and the assumption of a constant current $i\left(t_{c}\right)$ are two different issues. The former and the latter are expressed in Equations (12) and (13) in our paper (see p. 11916). From Equation (12), it is obvious that $t_{c}$ and the existence of a steady-state growth of PAOX splits the integral $\int_{t_{0}}^{t}\Pi i\left(t\right)dt$ into the two parts $\int_{t_{0}}^{t_{c}}\Pi i\left(t\right)dt$ and $\int_{t_{c}}^{t}\Pi i\left(t\right)dt$, which both represent the entropy production at different stages of the reaction. Both parts contain the term $\Pi i\left(t\right)$, where Π represents the derivation of intrinsic entropy with respect to time and charge. The main difference between these two parts is that the latter has to have a minimum entropy production >0 [4,5,7]. This extremum principle is applicable because the system is in a steady state at time point $t_{c}$. Both terms Π and $i\left(t\right)$ have a multiplicative dependence on each other. Thus, irrespective of the shape of the function $i\left(t\right)$ (i.e., the current–time curve), the necessary condition is always that $\int_{t_{c}}^{t}\Pi i\left(t\right)dt$ has to be minimized. It follows that the time point $t_{c}$ is not necessarily connected to a specific change in the current–time curve because the multiplicative dependence of Π and $i\left(t\right)$ allows an infinite amount of solutions. This is because every function $i\left(t\right)$ can be compensated by a specific function Π to reach the same minimum value of entropy production. Therefore, $i\left(t\right)$ can show in principle every shape while $t_{c}$ still exists.

Furthermore, MP mentioned in his review on p. 53 (see [1] Figure 30E) that “*the discussed mathematical model is not applicable due to the constant exponential decrease in j with time*” (herein, j denotes the current density). In addition to the fact that MP does not explain what part of the mathematical body is meant here, his statement is not true, because $i\left(t_{c}\right)$ can just be replaced by an inverse exponential function. In fact, in this case, p will then presumably not be the outcome of the mathematical framework. As mentioned above, we did not claim the general validity of our theory for all specific experimental results. Therefore, with regard to the comparability with the experiment, we pointed out in our paper that the occurrence of the parameter p at the end of the derivation indicates that our derivation *could* reflect experimental reality (see our paper p. 11914). Moreover, if the special assumption of a stationary and constant current $i\left(t_{c}\right)$ has to be applied to derive the parameter p, this says more about p than about our derivation, namely, that p is only applicable in such special situations from the theoretical point of view. This fact was not discussed by MP at all.

In connection with this issue, MP also states that our model “*does not formulate the sufficient conditions for stable PAA formation in a broader theoretical sense*” (see [1] p. 50). As expressed by us (see our paper p. 11916), the term $P_{c}+P_{0}$ (which is connected to the entropy production of the system) can be divided into parts before $t_{c}$ (non-stationary part of the reaction; $P_{0}$) and after $t_{c}$ (stationary part of the reaction; $P_{c}$). Hence, whatever MP means by “a broader theoretical sense”, we clearly depict that the term $P_{0}$ “*can be interpreted as a “history term” of the system, because it consists of information about the process before the constant current is established and where the pore habitus is grounded*” (see our paper p. 11916). In addition, the “general evolution criterion” [4] was the starting point of our derivation (see our paper p. 11916 Equation (10)). Thus, the conditions for PAA formation are already implied in this term and therefore also in our theory. The “general evolution criterion” is due to the second derivative of the internal entropy with respect to time. As we point out in our paper, our results are according to the usage of the entropy production (i.e., the first derivative of internal entropy with respect to time). Hence, the aim of our paper was not to derive theoretical reasons for the stability of the hexagonal cells, but to derive the factor p, which are mathematically two different issues. This fact was not mentioned by MP.

If one follows MP’s statements, he identifies our supposed “*next incorrect assumption*” (p. 51 [1]), which presumably follows from disregarding the water oxidation process: “the concentration of $H^{+}$ is minimized because of repulsion forces” at the position of the anode $x_{0}$ (p. 11917 in our paper). It is generally known that charged surfaces repel ions with the same charge. As the anode and $H^{+}$ both possess a positive charge, the latter are repelled from the former. Hence, the assumption of a minimum concentration of $H^{+}$ at $x_{0}$ is implied by simple electrostatics. There is no argument given by MP as to why our assumption should be incorrect. Depending on the studied mechanism, the water oxidation mechanism is assumed to directly take on top of the barrier layer (see, e.g., [12]) (i.e., not at $x_{0}$) or near the anode [1,3]. However it is in a real experiment, a maximum or minimum concentration of $H^{+}$ ions at the anode leads to the same mathematical result, i.e., the first derivation of the $H^{+}$ concentration with respect to *x* is 0. As the factor p has no connection to the mechanism of electroconvection proposed by MP but is only an assumed analogy and comparability to the Rayleigh–Benard instability [3], the appearance of p in our theory implies no preference for the evolution mechanism of anodic alumina and the conditions associated herein.

In the following, MP argues that “*charge carrier currents coupled with fluid flows can be also observed in purely physical systems without any chemical reactions. Thus, the Al oxidation reaction can only be of secondary importance for the emergence of self-organized flows in this case*” (see p. 51). In addition to the fact that MP does not explain or categorize in any way for the reader what is meant by this primary or secondary effect that he mentioned, he does not give a reference or an example for his argument. The occurrence of an ion source (the Al-oxidation reaction in our case) is a condition for the existence of ions and thus, for their flow towards the electrolyte bulk in our theory. Therefore, the oxidation reaction itself only provides the ions to inject charge into the system and form the hexagonal flow pattern. This so-called charge injection is the same principle as discussed in the references cited by MP on p. 42 of his review, where he mentioned the appearance of convective cells in dielectric liquids exposed to an electric field [6,13]. The absence of a chemical reaction in a purely physical system (whatever this is) is therefore no argument against our ansatz.

On p. 52 (5.), MP mentioned and wrote that the condition of initially set hexagonally arranged pores without explanation of their emergence which should be “*practically the same approach as was used in the Parkhutik-Shershulsky model*” was criticized “*by some other authors as unfounded*” (an approximately equal statement by MP can be also found at p. 35). Here, MP cites Li et al. [14] as “*some other authors*” but reproduces the content of their paper deviating from the actual content. Originally Li et al. indeed wrote about the Parkhutik–Shershulsky model (p. 2478 Li et al. [14]):

“*This theory maintained the assumed initial hemispherical shape for a pore bottom ‘…’ but did not explain how this shape started, nor did it explain the hexagonal ordering of the pores*”.

The original wording of the citation is, therefore, not the same as MP represents it.

Continuing with commentary given by MP on our paper, he wrote on p. 52 (6) that the resistivity of the cell is not constant and, therefore, Ohm’s law cannot be applied in this case. This points towards our usage of Ohm’s law to calculate the specific conductivity of a volume directly at the cathode ($\sigma_{\lambda_{c}}$). As this is done on the basis of the assumption that the process is in a stationary state with a constant current $i\left(t_{c}\right)$ (i.e., a constant amount of $H^{+}$ ions reduced per unit time at the cathode), potential (potentiostatic process) and current are both constant and so is the resistivity, i.e., Ohm’s law is applicable in this case. This is an ideal situation, but that is why we call it a modeling. Furthermore, the linear relationship of potential and current (i.e., amount of $H^{+}$ ions reduced per unit time at the cathode), namely, Ohm’s law is an inevitable condition for MP’s factor p to appear from the mathematical body in the context of our theory. Therefore, if Ohm’s law is not met in the system, p does not apply, too.

In the following, MP identifies that our so-called “*mechanism of charge reduction*” is invalid and that it “*violates the charge conservation principle and two Faraday laws at the same time*” (see [1] p. 52). As can be easily followed, we set the overall neutrality condition of the system (p. 11918, Equation (32) in our paper) as the starting point of our considerations, which is also described in our text.

We stated that

(3) $$c_{red,H^{+}}=c_{Al,H^{+}}+\sum_{A}c_{A,H^{+}}$$

where $c_{red,H^{+}}$ is the complete amount of reduced $H^{+}$, $c_{Al,H^{+}}$ is the amount of $H^{+}$ needed to compensate the charge of $Al^{3+}$ ions and $\sum_{A}c_{A,H^{+}}$ is the amount of $H^{+}$ needed to compensate the charge of anions incorporated in the film. As the only possible reaction to compensate positive charge in the system is the reduction of $H^{+}$ ions at the cathode (see our paper p. 11914 reaction 3 and 4 and text below), the implementation of all regarded positive excess charges into $c_{red,H^{+}}$ satisfies the neutrality condition, as done by us in Equation (32). This also disproves MP’s argument that “*the mechanism suggested by the authors of the mathematical model from Ref. [150] does not seem reasonable due to contradiction to the charge conservation principle*” (see [1] Figure 32), because the charge neutrality of the studied system was established from the outset. In addition, our arguments also show that Faraday’s laws are satisfied because the amount of substance consumed or produced during the electrolytic reaction is directly proportional to the charge flowing through the electrodes, as represented by reaction 3 and 4 and Equation (32).

Again, in this context, MP states that “*the transfer of anions into PAA is supposed to create excess positive charge within the solution*” and that “*no mechanism for negative charge compensation in PAA was proposed*” (p. 52 [1]), so the PAA layers must be charged negatively. We wrote in our paper “*To compensate the excess positive charge which evolves by the loss of anions in the bulk, an additional amount of $H^{+}$ ions have to be reduced at the cathode*” (see our paper p. 11918). This statement can only be “misleading” (see [1] p. 53 (7)) if one does not take for whatever reason the context and conditions given and defined by us in our paper into account. Anions have a negative charge, which is compensated by associated $Al^{3+}$ ions in the film (which are again, of course, the source for electrons to reduce $\sum_{A}c_{A,H^{+}}$ at the cathode), which is implied by the definition of overall charge neutrality as explained above but not explicitly formulated in our paper. In addition, no other anions that originate from the solution (e.g., $OH^{-}$) are considered in our theory because only acid anions are mentioned to flow towards the anode.

At the end of chapter 7.1 (see [1] p. 54), MP again uses terms like “*primary factors*” and “*secondary factors*” that are not further defined by him. In that context, he claimed that “*Once primary factors are considered to their existing mathematical model, the resulting conclusions become rather arbitrary*”. In addition to the fact that MP did not discuss or in any way disprove the mathematical basis or model of our theory at any point in his writing, he does not explain why the results should become arbitrary.

As shown above, MP’s arguments draw a distorted picture of our article. Moreover, his discussion did not mention the implications of our results for his own work, namely, the p-factor and his electroconvective theory. The derivation of the p factor from a mathematically and physically correct framework shows how p is related to the entropy production of a system that possesses a stationary state including a constant current that results from non-stationary conditions. It was shown by us that the entropy production and p solely depend on migration and that diffusion and convection are negligible.

This is in sharp contrast to the assumption of MP in which he states that the flows that form the hexagonal cells originate from two “*competing factors-namely, electromagnetic forces (Coulomb attraction) and the oppositely directed diffusion upon reaching a critical concentration gradient near the anode surface*” (see [1] p. 41). MP does not question his original assumptions in the light of our theory. However, this would be scientifically quite interesting since the linear dependence of p on the potential difference and the absence of a concentration gradient or velocity as shown in Equation (1) underlines our results. Therefore, it would be scientifically necessary to explain why the chemical model based on diffusion and Coulomb attraction [3] does not correlate with p, which has its mathematical–physical origin demonstrably only in migration [2].

Interestingly, this necessity is underlined by MP himself, who wrote on p. 48 of his review: “*The main criticism of the electroconvective model of PAA formation currently concerns the semi-empirical character of the criterion P, which defines the critical conditions for porous structure appearance*”. Furthermore: “*However, formal modeling still can provide a valuable ‘sanity check’ for the semi-qualitative theory. That is, it can test the correctness of the theoretical interpretation of empirical results by considering specific underlying phenomena. New results may reinforce the existing theoretical framework or extend it, but they may also suggest the necessity of a fundamental rethinking*”.

Therefore, the question arises how p can be associated with phenomena such as diffusion [1,3] if it is not mathematically associated with its driving force, the concentration difference? If this association does not exist mathematically, what are the consequences for the “*Pashchanka theory*” (see [1] p. 41)? This would be worth elaborating on, especially with regard to the theoretical consistency of the chemical mechanism of PAOX growth proposed by MP. An in-depth discussion of this issue based on our theory, parts of which MP has discussed in detail over five pages, is missing from the review.

In general and independent from the criticism of our paper, MP’s review presents the basics of self-organization theory in short passages partly on the basis of selected references [6,7,15]. By doing so, a lot of claims and wordings like “*for the miracle called “spontaneous self-organization” to occur, a system must be stochastic*” ([1] p. 1), “*the next important property of self-organizing systems - namely, their nonlinearity*” ([1] p. 2), “*increment in randomness*” ([1] p. 6), “*to maintain the long-term stability of a dissipative self-organization process, the constant flow of energy through an open system must be supported*” ([1] p. 6), “*production of entropy in this case equals to the Rayleigh dissipation function R divided by absolute temperature T*” ([1] p. 51), or the “*constant energy flow principle*” ([1] p. 7) are given to the reader without explanation, definition, discussion, or literature citation. Hence, it is not possible to prove or disprove their meaning in a scientific way. Therefore, a clear explanation and definition of these terms is desirable in order to make their content scientifically accessible.

Furthermore, there are passages in the MP review which, in our opinion, are difficult to scientifically substantiate. On p. 17 of the review, MP wrote “*entropy deals specifically with irreversible, time-oriented processes*”, which is to be doubted considering that equilibrium processes are also assigned to a specific entropy value. Furthermore, this statement is originally part of a statement from Prigogine’s book, who wrote: *Entropy is an essential part of thermodynamics, the science that deals specifically with irreversible, time-oriented processes* (see [7] p. 17). MP in his review fails to cite this correctly but adopts the original quote without citation. Moreover and in the same context, MP wrote that Prigogine means entropy when he talks about the “*arrow of time*” (see [7] p. 19). This statement by MP can be refuted because Prigogine refers to Arthur Stanley Eddington [16] who, according to Prigogine, drew this conclusion.

In conclusion, we hope that the arguments and examples raised in this critical comment stimulate a further scientific discourse.
